# Chromatinized Protein Kinase C-θ: Can It Escape the Clutches of NF-κB?

**DOI:** 10.3389/fimmu.2012.00260

**Published:** 2012-08-28

**Authors:** Elissa L. Sutcliffe, Jasmine Li, Anjum Zafar, Kristine Hardy, Reena Ghildyal, Robert McCuaig, Nicole C. Norris, Pek Siew Lim, Peter J. Milburn, Marco G. Casarotto, Gareth Denyer, Sudha Rao

**Affiliations:** ^1^Discipline of Biomedical Sciences, Faculty of Applied Science, The University of CanberraCanberra, ACT, Australia; ^2^The John Curtin School of Medical Research, Australian National UniversityCanberra, ACT, Australia; ^3^School of Molecular Bioscience, The University of SydneySydney, NSW, Australia

**Keywords:** PKC-theta, microRNA, chromatin, T cells, signaling kinase, immune response gene, NF-κB, nuclear PKC-theta

## Abstract

We recently provided the first description of a nuclear mechanism used by Protein Kinase C-theta (PKC-θ) to mediate T cell gene expression. In this mode, PKC-θ tethers to chromatin to form an active nuclear complex by interacting with proteins including RNA polymerase II, the histone kinase MSK-1, the demethylase LSD1, and the adaptor molecule 14-3-3ζ at regulatory regions of inducible immune response genes. Moreover, our genome-wide analysis identified many novel PKC-θ target genes and microRNAs implicated in T cell development, differentiation, apoptosis, and proliferation. We have expanded our ChIP-on-chip analysis and have now identified a transcription factor motif containing NF-κB binding sites that may facilitate recruitment of PKC-θ to chromatin at coding genes. Furthermore, NF-κB association with chromatin appears to be a prerequisite for the assembly of the PKC-θ active complex. In contrast, a distinct NF-κB-containing module appears to operate at PKC-θ targeted microRNA genes, and here NF-κB negatively regulates microRNA gene transcription. Our efforts are also focusing on distinguishing between the nuclear and cytoplasmic functions of PKCs to ascertain how these kinases may synergize their roles as both cytoplasmic signaling proteins and their functions on the chromatin template, together enabling rapid induction of eukaryotic genes. We have identified an alternative sequence within PKC-θ that appears to be important for nuclear translocation of this kinase. Understanding the molecular mechanisms used by signal transduction kinases to elicit specific and distinct transcriptional programs in T cells will enable scientists to refine current therapeutic strategies for autoimmune diseases and cancer.

## Introduction

In the past, the nuclear role of Protein Kinase C (PKC) has been dominated by the cytoplasmic signaling role of this kinase family. A complex series of molecular events is initiated upon stimulation of the TCR and CD28 co-receptor. This results in the selective activation of the novel PKC family member, PKC-θ, which activates transcription factors such as NF-κB, ultimately leading to induction of a distinct cohort of immune response genes (Sun et al., 2000; Isakov and Altman, 2002). However, recent studies have demonstrated that signaling molecules and kinases play a key role in the nucleus under activating conditions (Martelli et al., 1999; Passalacqua et al., 1999).

An emerging concept is that “chromatin structure” forms a novel platform for signal transduction. One elegant example of this is the activation of the stress-induced MAP kinase Hog1 that involves recruitment of this protein to the chromatin of most osmo-inducible genes in yeast (Pascual-Ahuir et al., 2006; Pokholok et al., 2006; Proft et al., 2006). Genome-wide analysis showed the binding pattern of Hog1 was not only within the promoter but also the transcribed regions of osmotic stress responsive genes (Proft et al., 2006). Several kinases have now been shown to occupy both the promoters and active transcribed regions of genes they activate (Pokholok et al., 2006), and this has helped to revolutionize our view of how signaling pathways regulate gene expression.

Recently, PKC-β_1_ was shown to associate with chromatin, phosphorylate threonine-6 on histone H3, and influence the action of the histone demethylase, LSD1 (Metzger et al., 2010). Analogous to these studies, we found that the PKC-θ enzyme tethers to chromatin and appears to be an integral component of transcription complexes, where it functions as a structural adaptor or locally phosphorylates other chromatin-associated proteins (Sutcliffe et al., 2011). In certain cohorts of inducible genes in T cells, nuclear PKC-θ intimately interacts with chromatin by forming an active transcription complex with RNA polymerase II, MSK-1, LSD1, and 14-3-3ζ (Sutcliffe et al., 2011). Specifically, we showed that nuclear PKC-θ is recruited in an activation-dependent manner to the proximal promoters of inducible T cell genes, such as *CD69*, *TNF-α*, *IFN-γ*, and *heparanase*, as well as to microRNA genes (Sutcliffe et al., 2011). One possible consequence of the association of activated signal transduction kinases with the genes they regulate is that it could provide a more efficient mechanism of controlling gene expression. Our findings revealed a model in which chromatin-anchored PKC-θ could regulate inducible T cell gene transcription by two opposing mechanisms. Firstly, via the direct tethering of PKC-θ to inducible genes to form an active transcription complex. Secondly, indirectly by docking onto the chromatin of microRNA genes that modulate key repressor proteins that regulate cytokines. Ultimately, the balance of both these processes allows appropriate levels of inducible gene transcript to be maintained in activated T cells.

Here, we extend upon our recent observations describing PKC-θ as a novel chromatin-associated enzyme, by identifying two distinct NF-κB-containing motifs. The first may facilitate the assembly of the PKC-θ active complex at protein coding genes and the second enables negative regulation of microRNA genes. Finally, we have identified a new nuclear localization sequence for PKC-θ that may help to delineate the nuclear and cytoplasmic functions of this important kinase.

## Results and Discussion

### Identification of a novel nuclear localization signal for PKC-θ

Many proteins that are targeted to the nucleus contain a canonical nuclear localization signal (NLS) and indeed a putative NLS domain has been described for PKC-θ (DeVries et al., 2002). Alternatively, proteins can bind to NLS-containing proteins in order to enter the nucleus. Preliminary computational analysis using publically available yeast two-hybrid screens revealed candidate proteins that could facilitate the transport of PKC-θ into the nucleus, namely AKT1, HABP4, CHD3, and TCLA1. Based on our preliminary studies, these two mechanisms are unlikely to solely account for the existence of nuclear PKC-θ.

Chuderland et al. (2008) have demonstrated that the Serine-Proline-Serine (SPS) sequence in ERK2 is phosphorylated following cellular stimulation and that this is essential for nuclear translocation. Other signaling molecules, such as SMADS, MEK, and p53 use the same mechanism. Indeed, mutation of SPS to APA (which cannot undergo phosphorylation), greatly reduced nuclear translocation of ERK2, whereas mutation to EPE (which mimics a phosphorylated SPS sequence), resulted in highly upregulated nuclear translocation of ERK2 (Chuderland et al., 2008). SPS phosphorylation was required for the interaction of ERK2 with the nuclear import protein, Importin 7 (Chuderland et al., 2008). Although the specific kinases responsible for SPS phosphorylation were not identified, S/T-P-S/T was suggested to be a general nuclear translocation signal for NLS-independent translocation of signaling molecules into the nucleus.

**Figure 1:**
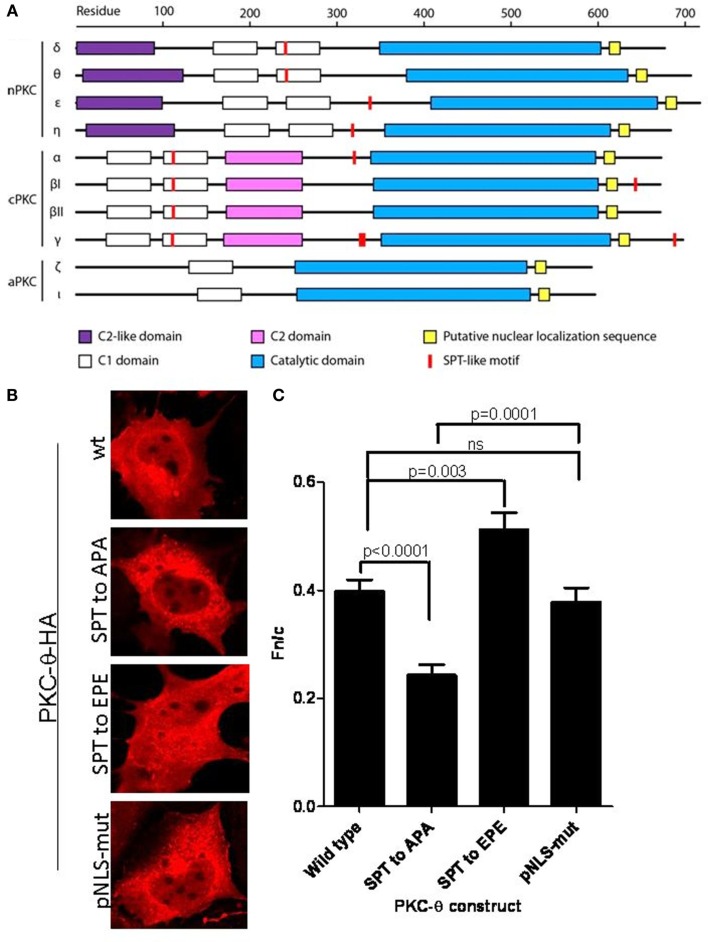
**SPT^243^ is the minimum motif for nuclear localization of PKC-θ regulated by phosphorylation at S^241^ and T^243^**. **(A)** SPT-like motifs and nuclear localization sequences (NLS) were displayed relative to the domain organization of PKC members (nPKC: δ, θ, η, ε; cPKC: α, βI, βII, γ; and aPKC: ζ, ℩, or λ). SPT-like motifs are depicted in red and the locations of NLS are shown in yellow. The C2-like domain (purple) is characteristic of nPKCs, while cPKCs share the calcium binding C2 domain (pink). Both nPKC and cPKC isoforms possess the C1 domain (white), that is composed of C1a and C1b. In comparison, aPKC isoforms only contain C1a of the C1 domain. All PKC family members contain a C-terminal catalytic domain (blue). **(B)** The full length PKC-θ wild type gene sequence and its derivatives wherein putative phosphorylation sites at S^241^ and T^243^ were mutated to either the non-phosphorylatable alanine (SPT to APA) or the phosphomimetic glutamine (SPT to EPE), were cloned into the pTracer-CMV vector in frame with a C-terminal HA-tag. The vector also codes for GFP, which is translated independent of the insert and serves as an internal marker for transfected cells. Subconfluent cultures of Cos-7 cells were transfected and subsequently the fixed cells were probed with rabbit antibody to HA-tag, followed by secondary antibody to rabbit immunoglobulins conjugated to Alexa-Fluor 568. Localization of expressed PKC-θ was studied with confocal laser scanning microscopy as detailed in the methods. Representative images for each construct are shown. **(C)** *F*_n/c_ values for each construct are shown in **(C)**, with significant differences between datasets indicated. Data shown are mean ± SEM, *n* > 15 for each dataset.

Thus, in our experiments we searched the sequences of the human PKC family members for S/T-P-S/T (SPT-like) motifs and corresponding NLS sequences previously described (DeVries et al., 2002). Figure 1A showed that out of all the possible residue combinations for this motif, the sequence “SPT” was the most common. Additionally, all nPKC and cPKC isoforms contain at least one SPT-like motif (Figure 1A). In contrast, aPKC isoforms do not contain any SPT-like motifs. Interestingly, there is an SPT-like motif in the second C1 domain, C1b, in all cPKC isoforms, and nPKC δ and θ (Figure 1A). The nPKC isoforms ε and η do not contain this conserved motif, instead they contain the sequence VPT at this location in C1b. However, both these isoforms have an additional SPT-like motif in between C1b and the catalytic domain (Figure 1A). This could indicate that there has been a compensatory mutation in these two isoforms. Two additional SPT motifs were found in PKC-γ, one of which is repeated (SPSPSPT). Additionally, there is a C-terminal TPT motif present in PKC-βI that is not in βII (Figure 1A). Certain SPT-like motifs in PKC-η, α, and βI, have been experimentally shown phosphorylation sites, and others have been predicted to be phosphorylated (see “Table in Materials and Methods”).

We designed two mutants of the SPT motif within the full length PKC-θ, expressed them as HA-tagged fusion proteins, and assessed the distribution of each PKC-θ mutant construct following transfection into Cos-7 cells. Our results show that mutation of the SPT motif in PKC-θ to APA, which cannot be phosphorylated, resulted in a significant decrease in the nuclear localization of this kinase (Figures 1B,C). Mutation of this motif to EPE, a change that mimics constitutionally phosphorylated PKC-θ, led to an increase in nuclear localization (Figures 1B,C). Interestingly, mutation of the putative NLS sequence (pNLS-mut) did not significantly alter cytoplasmic-nuclear distribution of this kinase in Cos-7 cells (Figures 1B,C). These mutants will be a valuable tool to determine how essential nuclear localization of PKC-θ is to inducible gene regulation.

Taken together, our findings suggest that PKCs belong to the class of signaling kinases whose nuclear transportation and role may be accomplished through this alternative NLS motif. Further studies will be required to determine which kinases mediate the phosphorylation of this motif in T cells and to what extent the NLS and SPT sequences of PKC-θ participate in mediating nuclear shuttling of this kinase in different cell types and activation states.

### PKC-θ is active in both cytoplasmic and nuclear fractions

**Figure 2:**
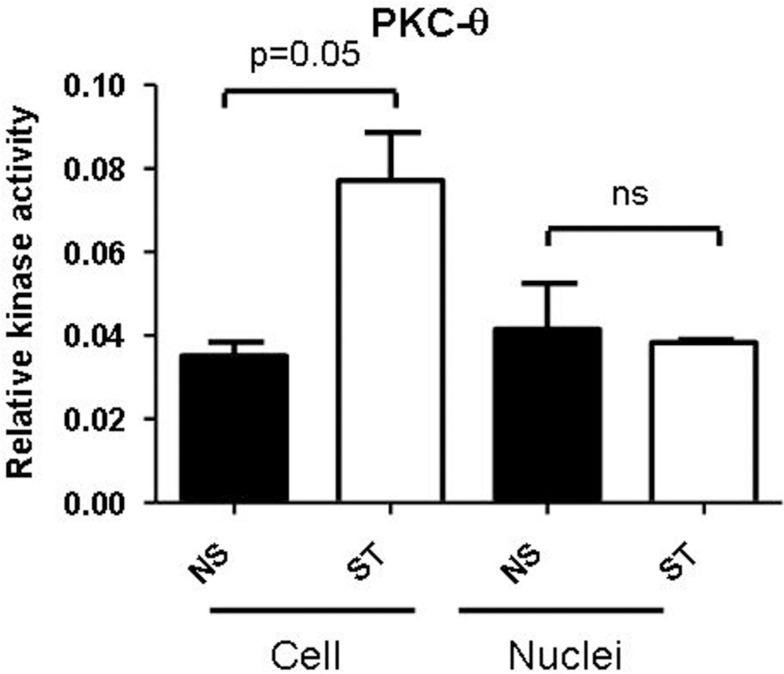
**PKC-θ activity in T lymphocytes**. Jurkat T cells were either non-stimulated (NS) or stimulated (ST) with PMA/CaI for 4 h. The PKC-θ specific antibody was used to immunoprecipitate PKC-θ from either whole cells or nuclei from both NS and ST treated Jurkat T cells. PKC ELISA-based kinase assays were performed on these PKC-θ fractions and absorbance was measured at 450 nm. Data is plotted as relative kinase activity compared to the negative control that did not have any antibody. Data is representative of the mean ± SE of three independent experiments and statistical significance was determined by a two-tailed paired *t*-test using GraphPad Prism 5.03.

Protein Kinase C-theta clearly has distinct functions in both the cytoplasm and nucleus (Sutcliffe et al., 2011). However, the relative activity of this isoform in these two compartments is unknown. Our PKC-θ isoform specific kinase assay revealed that this kinase is active in both the cellular and nuclear compartments in resting and activated T cells (Figure 2). Interestingly, cytoplasmic PKC-θ activity increased following T cell activation, whereas there was no significant induction in the nuclear fraction (Figure 2). Although there is a lack of induction in nuclear PKC-θ activity following T cell stimulation, it remains to be determined how activity may influence the function of this kinase when it is tethered to chromatin. Indeed, our preliminary results indicate that the active form of this kinase (phosphorylation of serine-695) is much more transient on chromatin than the occupancy of PKC-θ in the non-phosphorylated form (Sutcliffe et al., 2011). Ultimately, the structure and function of chromatin-tethered PKC-θ needs to be elucidated to further understand how this kinase mediates gene regulation.

### An NF-κB transcription factor module exists within PKC-θ bound genes in human T cells

The mechanisms that control the recruitment of signaling kinases to particular chromatin regions remain elusive, although the binding location of PKC-θ at specific regions of DNA and on selected genes may provide a clue to both its function and mode of recruitment (Edmunds and Mahadevan, 2006). Based on various occupancy patterns across genes, Pokholok et al. (2006) present several models of how signaling kinases can be recruited and associate with the genome. Kinases could be recruited to regulatory regions of inducible genes initially by specific marks such as PTMs on histones or by specific transcription factors in response to T cell activation. Once recruited, these kinases could associate with the transcriptional machinery or with remodeling complexes.

**Figure 3:**
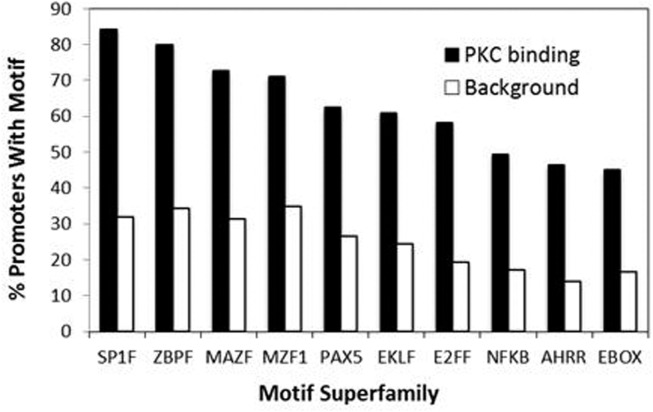
**Motif superfamilies identified within promoters of PKC-θ bound genes**. A greater proportion of the promoters of the PKC-bound genes had motifs for members of the SP1F, ZBPF, MAZF, MZF1, PAX5, EKLF, E2FF, NFKB, AHRR, and EBOX motif superfamilies, when compared to a background set of promoters. The representative members of the superfamilies shown are SP1_01 (SP1F), ZNF219_01 (ZBPF), MAZR_01 (MAZR), MZF1_01 (MZF1), PAX5_01 (PAX5), EKLF_01 (EKLF), NFKAPPAB_01 (NFKB), E2F_03 (E2FF), AHRARNT_02 (AHRR), and MYCMAX_03 (EBOX).

**Figure 4:**
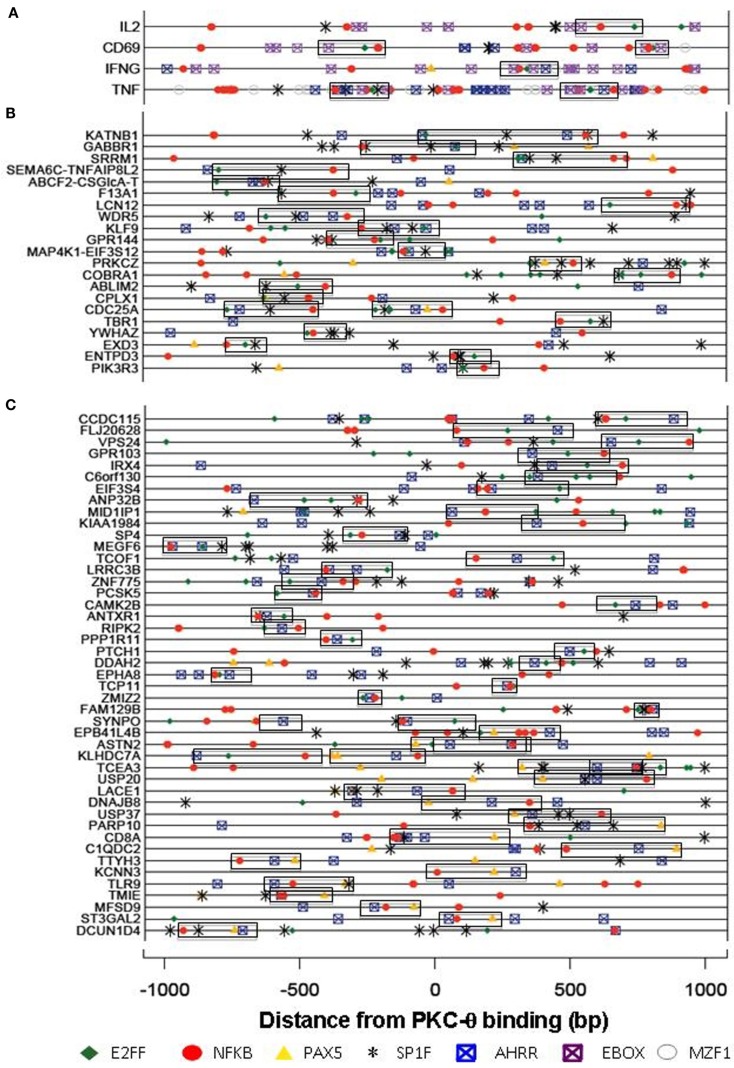
**Transcription factor binding motifs in the PKC-bound sequences**. Motifs for members of the NFKB (red), E2FF (green), PAX5 (orange), AHRR (blue), EBOX (purple), MZF (gray), and SP1F (black) superfamilies were identified by their Position Weight Matrices. PKC binds four inducible genes [+150 bp downstream from the transcription start site **(A)**], in promoter sequences **(B)** or within genes **(C)**. All motifs within a superfamily are shown for **(A)** while only NFKAPPAB_01 (NFKB), E2F_03 (E2FF), AHRARNT_02 (AHRR), PAX5_01 (PAX5), and SP1_01 (SP1F) are shown for **(B)** and **(C)**. Commonly co-occurring motifs are boxed together. NFKB_SP1F_E2FF commonly co-occur in the promoter regions **(B)**, while the NFKB_E2FF_AHRR and NFKB_AHRR_PAX5 combinations are significantly over-represented in the within-gene PKC binding regions **(C)**.

Our recent genome-wide analysis identified many novel PKC-θ targeted genes that might be implicated in Th1 cell development, differentiation, apoptosis, and proliferation (Sutcliffe et al., 2011). To examine the transcription factor candidates that may be responsible for recruiting PKC-θ to target genes, we undertook bioinformatic analysis of our ChIP-on-Chip data. Forty-nine over-represented transcription factor binding motifs were identified in the promoters of the PKC-θ binding genes. These motifs belonged to 10 superfamilies ZBPF, MAZF, MZF1, SP1F, EBOX, E2FF, EKLF, PAX5, NFKB, and AHRR (Figure 3). The promoters of *IL-2*, *CD69*, *TNF-α*, and *IFN-γ* (all strongly transcriptionally regulated during T cell activation) were examined for these motifs (Figure 4A). NF-κB motifs were located near E2FF and EBOX family members in all four genes (Figure 4A).

In other PKC-θ binding genes, the area around the PKC-θ binding site was analyzed for commonly occurring combinations (co-regulatory motifs, CRM) of three motifs, where the most frequently occurring superfamily member was used and the frequency of three motifs occurring within 300 bp of each other was calculated. MYCMAX_03 (EBOX) and AHRARNT_02 (AHRR) commonly occurred together and this was attributed to the similarity of their matrices. The combination NFKB_SP1F_E2FF occurs in these promoter regions significantly more than expected by chance (Figure 4B), whereas the NFKB_E2FF_AHRR and NFKB_AHRR_PAX5 combinations are significantly over-represented in the within-gene PKC binding regions (Figure 4C). The PAX5 DNA binding motif is bound by both Pax5 and Pax-9 of which only Pax-9 is expressed in Jurkat cells. ChIP-seq data for GM12878 and K562 cells (Lee et al., 2012) available in the UCSC Hg18 genome browser suggests that the AHRR sites may actually be bound by c-Myc and/or Max. This study also showed that NF-κB can bind near many of the predicted sites (Lee et al., 2012).

Currently it is unknown what targets chromatin-remodeling factors and signaling kinases, such as PKC-θ, to specific chromatin regions. This data provides a clue that a novel NF-κB transcription factor module may be involved in tethering PKC-θ to chromatin in T cells.

### NF-κB is a prerequisite for the tethering of the PKC-θ active transcription complex

The NF-κB family of transcription factors encompasses a heterogenous set of inducible proteins, including NF-κB1 (p50 and precursor p105), NF-κB2 (p52 and precursor p100), RelA (p65), RelB, and c-Rel (May and Ghosh, 1998; Caamano and Hunter, 2002; Kane et al., 2002). The phosphorylated NF-κB dimer translocates into the nucleus where it binds to DNA at cognate κB sites in promoters and enhancers to control transcription of various NF-κB dependent genes (Donovan et al., 1999; Rayet and Gelinas, 1999; Seetharaman et al., 1999; Das et al., 2001). These genes are expressed in a cell- and tissue-specific manner and this provides an additional level of regulation. Generation of mice deficient in, or having mutated forms of, the different members of the NF-κB family has helped to delineate characteristics of each member in various immune responses and their role in disease (Kontgen et al., 1995; Ghosh et al., 1998; Rao et al., 2003).

**Figure 5:**
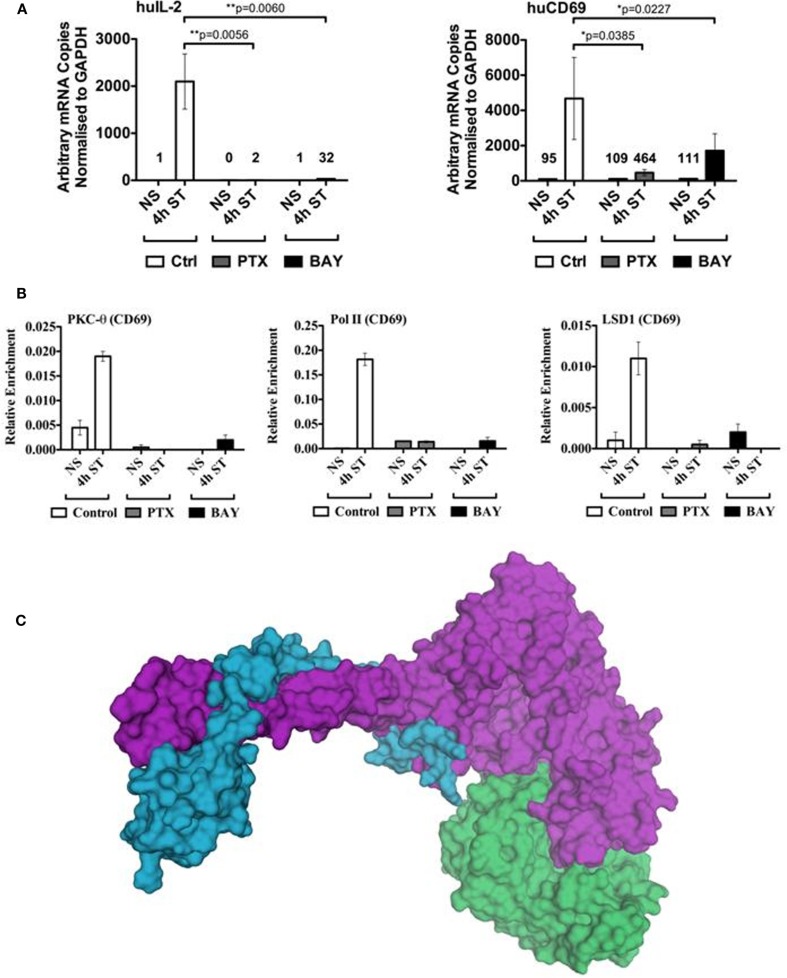
**Binding of the PKC-θ active transcription complex to the *CD69* promoter appears dependent on NF-κB**. **(A)** Resting Jurkat T cells were pre-incubated with either Pentoxifylline (PTX), Bay, or left untreated (control) for 1 h. Cells were then left non-stimulated (NS) or activated with PMA/CaI (ST) for 4 h. Total mRNA was isolated for cDNA reverse transcription. Gene specific Taqman^®^ expression assay was used to detect the transcript levels of human *IL-2* and *CD69*. Data is expressed as arbitrary mRNA copies normalized to *GAPDH*. Data represent the mean ± SD of four independent experiments. Statistical significance between the activated control and inhibited samples was determined by a two-tailed paired *t*-test using GraphPad Prism 5.03. PKC-θ, Pol II, and LSD1 ChIP experiments were performed on the samples described in **(A)**. **(B)** Sybr-green real-time PCR was used to detect the relative enrichment of these proteins across the *CD69* promoter (−214 to −52 relative to the transcription start site). **(C)** The catalytic domain of PKC-θ (green, pdb code 2JED) is modeled interacting with the LSD1-CoREST complex (purple and blue, respectively, pdb code 2IW5). In this model the peptide-binding groove of PKC-θ contacts the SWIRM and amine oxidase domains of LSD1.

To functionally validate that NF-κB may be involved in the direct tethering of PKC-θ to chromatin, we utilized two well established NF-κB inhibitors. Pentoxifylline directly inhibits c-Rel activity, preventing its nuclear recruitment in CD4^+^ T cells (Wang et al., 1997), whilst Bay disrupts the phosphorylation of IκB, resulting in the inappropriate cytoplasmic retention of NF-κB (Garcia et al., 2005). Consistent with previous findings, pretreatment of Jurkat T cells with either of these NF-κB inhibitors significantly abolished inducible gene transcription of two key immune response genes, *IL-2* and *CD69* (Figure 5A).

Next, we examined whether recruitment of PKC-θ, Pol II, or LSD1 were impaired in the inhibitor treated cells. Our chromatin immunoprecipitation (ChIP) results clearly show that the lack of NF-κB severely reduced assembly of the PKC-θ-containing active transcription complex (Figure 5B). These results further support our previous findings of the interdependency of PKC-θ and LSD1 (Sutcliffe et al., 2011), where both proteins are essential for the active complex to remain associated with chromatin. Ultimately to truly ascertain the specificity of the NFkB-PKC-θ axis at the level of chromatin structure, further experiments will be required with inhibitors that target other T cell transcription factors such as NFAT.

To examine whether PKC-θ and LSD1 have the potential to directly interact with each other, we modeled the interaction between PKC-θ and LSD1 using the ClusPro server (Kozakov et al., 2010) with the LSD1-CoREST crystal structure (pdb code 2IW5) and chain A from the crystal structure of the catalytic domain of PKC-θ (pdb code 2JED). This computational modeling revealed a high degree of complementarity between the LSD1 and PKC-θ surfaces at the putative interaction interface (Figure 5C). Interestingly, LSD1 is bound where the peptide substrate would bind PKC-θ. Functional studies are required to confirm there is a direct interaction between these two proteins.

### NF-κB relieves transcriptional repression of PKC-θ targeted microRNA, miR-200c, in human T cells

**Figure 6:**
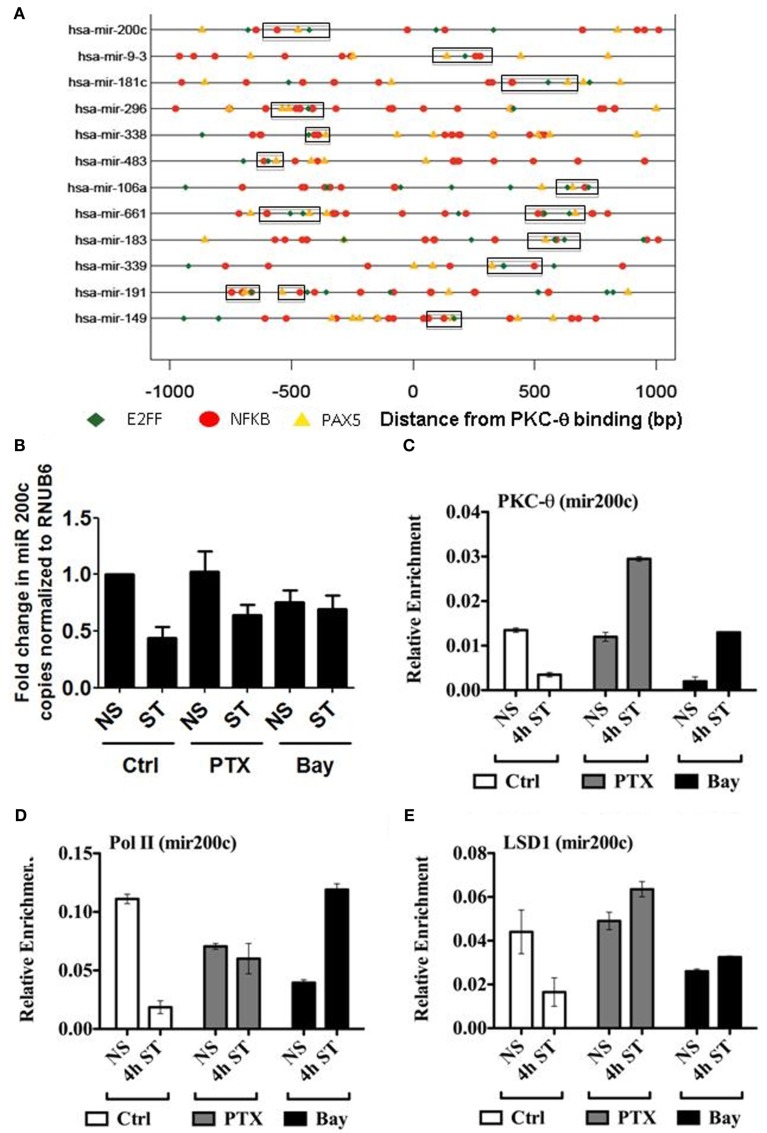
**PKC-θ binding to the miR-200c promoter is not impaired by the absence of NF-κB in T cells**. **(A)** Transcription factor binding motifs in the microRNA PKC-θ bound sequences. Motifs for NFKAPPAB_01 (NFKB, red), E2F_03 (E2FF, green), and PAX5_01 (PAX5, orange) were identified by their Position Weight Matrices. The three motifs commonly occur within 300 bp of each other near the PKC binding sites in microRNA genes. **(B)** TaqMan miR-200c microRNA cDNA was isolated from resting (NS) and 4 h PMA/CaI stimulated (ST) Jurkat T cells in the presence or absence of Pentoxifylline (PTX) or Bay. Data expressed as fold change in miR-200c relative to NS set to 1 and normalized to RNU6B. Data representative of the mean ± SE of three independent experiments. **(C)** PKC-θ, **(D)** Pol II, and **(E)** LSD1 ChIP were carried out on samples as described for **(B)**. Sybr-green real-time PCR was used to detect the relative enrichment of these proteins across the *miR-200c* promoter. ChIP data are expressed as relative enrichment and plotted as the mean ± SE of two independent experiments performed in duplicate.

Our recent ChIP-on-Chip data revealed that PKC-θ binds to both promoters and transcribed regions of genes, as well as to microRNA promoters that are crucial for cytokine regulation (Sutcliffe et al., 2011). Since PKC-θ regulates microRNAs in human T cells, we wanted to determine whether, like coding genes, an NF-κB-containing motif exists on these genes. To address this, a cohort of PKC-θ bound microRNAs were interrogated for the presence of the superfamily motifs identified in Figure 3. Interestingly, in these sequences a distinct combination of NFKB_PAX5_E2FF was commonly found (Figure 6A).

To functionally validate the importance of NF-κB in the transcription of microRNAs, we specifically investigated the behavior of miR-200c, which is dependent on PKC-θ recruitment (Sutcliffe et al., 2011). In addition, we again utilized the pentoxifylline and Bay inhibitors. As shown in Figure 6B, treatment with both these compounds resulted in increased transcription of miR-200c following T cell stimulation.

In parallel at the chromatin level, pentoxifylline and Bay increased recruitment of PKC-θ, Pol II, and LSD1 at the miR-200c promoter following T cell activation (Figures 6C–E). These ChIP data further support the interdependence of these proteins and implicate NF-κB in the regulation of miR-200c. However, in contrast to the coding genes so far examined, the PKC-θ active transcription complex appears to be recruited to microRNAs independently of NF-κB to relieve transcriptional repression.

## Conclusion

**Figure 7:**
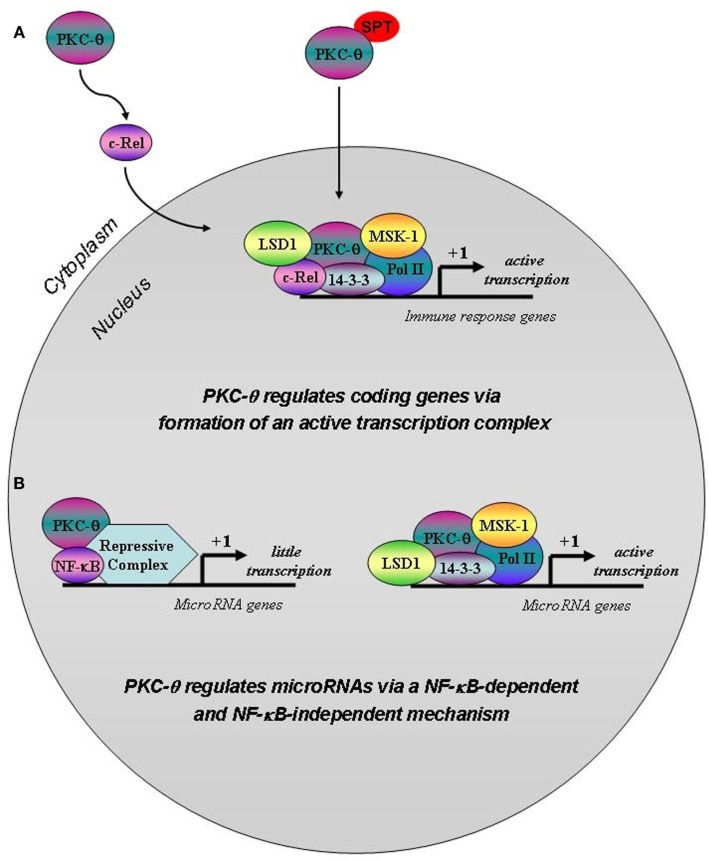
**Putative model for PKC-θ tethering to coding vs. microRNA genes in T cells**. **(A)** T cell activation induces a complex signaling cascade that involves PKC-θ, which provokes the activation and nuclear translocation of the transcription factor c-Rel. This event ultimately results in the binding of c-Rel to a unique transcription factor module at gene regulatory regions. We propose that c-Rel then initiates the recruitment of PKC-θ that may enter the nucleus via a phosphorylatable SPT sequence and bind to chromatin, thereby allowing the formation of the active transcription complex on coding genes to enable chromatin accessibility and gene transcription. **(B)** Following T cell stimulation, there is a reduction in PKC-θ binding to chromatin at microRNA genes compared to resting T cells. Instead, NF-κB is recruited via a distinct transcription factor motif and may form part of a repressive complex on these genes to dampen gene transcription. When NF-κB is removed from this system, more of the active PKC-θ complex assembles on microRNA gene promoters thereby overcoming transcriptional repression.

The findings presented here provide a new dimension to the chromatin-associated role we previously described for PKC-θ (Sutcliffe et al., 2011). In this present study, we have shown that PKC-θ may be recruited onto the chromatin template via distinct NF-κB transcription factor modules depending on the gene context. Our results reveal the existence of an NF-κB dependent mechanism for the tethering of the active transcription complex comprising of at least PKC-θ, Pol II, and LSD1 on coding genes (Figure 7). We propose this mechanism involves PKC-θ mediated cytoplasmic activation of c-Rel leading to binding of this transcription factor on target genes (Figure 7). In the genes we have surveyed, these promoter complexes also comprise nuclear PKC-θ.

We have established that a novel SPT sequence must be phosphorylated in order for PKC-θ to be translocated into the nucleus (Figure 7). This event appears to be independent of its intrinsic kinase activity. The nuclear and cytoplasmic contributions of PKC-θ remain to be delineated, to unravel how they are coordinated and integrated to achieve the appropriate physiological response. Our findings of the existence of the novel SPT motif in novel and conventional PKC isoforms is exciting and will have a tremendous impact by allowing us to manipulate the nuclear role of PKC-θ. It will also be important to ascertain whether this nuclear phenomenon is intrinsic to all PKC family members depending on the cell type, activating signal, and differentiation state of the cell.

Several lines of evidence support our model of the involvement of c-Rel in mediating PKC-θ recruitment over other NF-κB family members: (1) the delayed kinetics of nuclear c-Rel translocation and activation following T cell stimulation closely correlates with the formation of the active transcription complex; (2) pentoxifylline is a c-Rel specific inhibitor that successfully abolishes the assembly of the PKC-θ complex; (3) our preliminary c-Rel knock-out studies suggest that c-Rel is essential for anchoring PKC-θ to chromatin (data not shown); and (4) both c-Rel and PKC-θ are essential for chromatin accessibility necessary for transcription of immune response genes (Rao et al., 2003; Sutcliffe et al., 2011). The notion that nuclear kinases work synergistically with NF-κB is not without precedence. Saccani et al. (2002) found that p38-dependent H3 phosphorylation marked selected promoters for increased recruitment of NF-κB leading to increased transcriptional activity. It remains to be determined whether PKC-θ is linked to the recruitment of histone modifying complexes or chromatin remodelers, such as SWI/SNF, as was previously demonstrated for p38α (Simone et al., 2004). In addition, other molecules of the NF-κB pathway such as IKK-α, NIK, and IKK1 have also been shown to have a nuclear role (Carlotti et al., 2000; Birbach et al., 2002; Anest et al., 2003; Yamamoto et al., 2003).

Our results also imply that NF-κB forms a repressive complex on microRNA genes by impeding the formation of the PKC-θ active transcription complex (Figure 7). Indeed we have identified that a unique NF-κB transcription factor module exists on microRNA genes which is distinct to that within PKC-θ bound coding genes. In this scenario when NF-κB is inhibited, the PKC-θ active transcription complex binds to target microRNAs relieving transcriptional repression. Future studies are required to decipher the make-up of this repressive complex, determine the order of recruitment during complex assembly and assess the phosphorylation status of chromatinized PKC-θ.

A fundamental question remains as to how PKC-θ tethers to chromatin at microRNA genes in the absence of NF-κB. One possibility being that PKC-θ is recruited via an epigenetic signature on histone tails or chromatin-associated proteins. For example, the 14-3-3ζ adaptor protein which we have shown to be part of the PKC-θ active complex (Sutcliffe et al., 2011) may act as an anchoring target for PKC-θ. Our findings provide a foundation for investigating the contribution of individual NF-κB family members in their ability to modulate nuclear PKC-θ mediated transcriptional events, both at coding genes and microRNA genes.

Our discovery of a molecular function of nuclear PKC-θ has challenged the previous concept that this kinase’s sole purpose is signal transduction in the cytoplasm following T cell activation. Given that PKC-θ has also been shown to play a pivotal role in the generation of auto-reactive effector T cells in autoimmune conditions and immunological memory (Marsland et al., 2005; Healy et al., 2006; Tan et al., 2006), understanding the molecular mechanisms used by PKC-θ to elicit specific and distinct transcriptional programs in T cells may provide new avenues for therapeutic drug design.

## Materials and Methods

### Cell culture

Human Jurkat T cells and mouse EL-4 T cells were cultured and stimulated with 24 ng/mL of PMA (Sigma) and 1 μM of CaI (Sigma) as previously described (Chen et al., 2005; Sutcliffe et al., 2009). For inhibitor studies, cells were pre-treated with 6 mg/mL pentoxifylline (Sigma), 15 μM Rottlerin (Calbiochem), 15 μM H89 (Sigma), or 5 μM Bay (Calbiochem) as specified for 1 h prior to stimulation. Cos-7 cells were cultured in DMEM high glucose medium with 10% heat inactivated FCS and antibiotics.

### Protein extract preparation

Both whole cell and nuclear extracts were prepared for PKC kinase assays. For whole cell extracts, cells were collected and washed once in 1 X PBS then resuspended in RIPA buffer [20 mM Tris pH 8.0, 15 mM NaCl, 5 mM EDTA, 1% Triton X-100, 0.2 mM Pefabloc (Roche), 1:200 Protease Inhibitor Cocktail Set III, EDTA-Free (Cat. No: 539134, Merck), 1:500 Phosphatase Inhibitor Cocktail Set V (Cat. No: 524629, Merck)] and incubated on ice for 15 min. Samples were centrifuged at 13,000 rpm for 15 min at 4°C and the supernatant was collected as whole cell extracts. For nuclear extracts, cells were collected and washed once in 1× PBS then resuspended in Nuclei Lysis Buffer A+ [10 mM Tris pH 7.4, 10 mM NaCl, 3 mM MgCl_2_, 0.1 mM EDTA, 0.5% NP-40, 0.2 mM Pefabloc (Roche), 1:200 Protease Inhibitor Cocktail Set III, EDTA-Free (Cat. No: 539134, Merck), 1:500 Phosphatase Inhibitor Cocktail Set V (Cat. No: 524629, Merck)] and incubated on ice for 5 min. Samples were centrifuged at 3000 rpm for 3 min at 4°C then the supernatant was removed. The remaining pellet was resuspended in Nuclei Lysis Buffer A− (as in Nuclei Lysis Buffer A but without NP-40) and centrifuged for 3000 rpm for 3 min at 4°C. The supernatant was removed and the nuclei were resuspended in SDS Lysis Buffer (1% SDS, 10 mM EDTA, 50 mM Tris, pH 8.1).

### PKC-θ kinase assay

Protein Kinase C-theta enzymatic activity was assayed using a modification of a chromatin immunoprecipitation method previously described (Sutcliffe et al., 2009). Briefly, whole cell and nuclear extracts were prepared and sonicated for 7 min. The sonicated samples were centrifuged at 14,000 rpm for 5 min to remove debris and the supernatant was collected. Samples were diluted with ChIP dilution buffer (Millipore) and incubated overnight at 4°C with 5 μg of anti-PKC-θ (sc212, Santa Cruz) and goat anti-rabbit IgG conjugated to agarose beads, affinity isolated antibody (Cat. No: A8914, Sigma-Aldrich). Samples were then processed with ChIP wash buffers (Millipore) and the Kinase Wash Buffer (Enzo Life Sciences). The beads were then resuspended in Kinase Assay Dilution Buffer (Enzo Life Sciences). The samples were loaded in duplicate wells on the PKC kinase activity plate and the assay was performed as per manufacturer’s guidelines (PKC kinase activity kit, Cat. No: ADI-EKS-420A, Enzo Life Sciences). The PKC kinase activity was measured at an absorbance of 450 nm on the Benchmark Plus™ Microplate Spectrophotometer (BioRad). PKC kinase activity was analyzed by firstly, subtracting the blank readings from the average of duplicate sample wells to correct for background absorbance. Then, the no antibody control well readings were subtracted from the corrected sample readings to give the relative kinase activity.

### Transfections and confocal microscopy

Within the full length PKC-θ wild type gene sequence the putative phosphorylation sites S^241^ and T^243^ were mutated to either the non-phosphorylatable alanine (SPT to APA) or the phosphomimetic glutamine (SPT to EPE) and were cloned into the pTracer-CMV vector in frame with a C-terminal HA-tag. This vector also codes for GFP which is translated independently of the insert and serves as an internal marker for transfected cells. Subconfluent cultures of Cos-7 cells grown on coverslips were transfected with purified plasmids using lipofectamine 2000 (Ghildyal et al., 2005) and fixed 24 h later with 4% paraformaldehyde in PBS, followed by permeabilization with Triton X-100 as described previously (Li et al., 2008). Fixed cells were probed with rabbit antibody to HA-tag (Sigma), followed by secondary antibody to rabbit immunoglobulins conjugated to Alexa-Fluor 568 (Life Technologies); coverslips were mounted on glass microscope slides with ProLong Gold antifade (Life Technologies). Localization of expressed PKC-θ was studied with confocal laser scanning microscopy as described previously (Ghildyal et al., 2009a). Single sections of 0.5 μM were obtained with Nikon x60 oil immersion lens using a Nikon C1 plus confocal system and NIS-Elements AR 3.2 software; the final image was obtained by averaging four sequential images of the same section. Digital confocal images were analyzed with ImageJ public domain software to determine the nuclear/cytoplasmic fluorescence ratio (*F*_n/c_), determined by using the equation: *F*_n/c_ = (*F*_n_ − *F*_b_)/(*F*_c_ − *F*_b_), where *F*_n_ is the nuclear fluorescence, *F*_c_ is the cytoplasmic fluorescence, and *F*_b_ is the background fluorescence (autofluorescence; Ghildyal et al., 2005, 2009a,b). Mann–Whitney non-parametric test (GraphPad Prism) was used to determine significant differences between datasets.

### Total RNA isolation and quantitative real-time PCR

Total RNA was extracted, converted to cDNA, and real-time PCR was performed as previously described (Sutcliffe et al., 2009). Human arbitrary *IL-2* and *CD69* transcript levels were detected using gene specific Taqman assays [*IL-2* (Hs00174114) and *CD69* (Hs00934033) Applied Biosystems]. Inducible gene transcript levels were normalized to the housekeeping gene, *GAPDH* (Hs99999905). MiR-200c and RNU6B microRNA assays were performed using the TaqMan^®^ MicroRNA Reverse Transcription Kit (ABI 4366596) and probes previously described (Sutcliffe et al., 2011).

### Chromatin immunoprecipitation assays

ChIPs were performed following the protocol supplied by Upstate Biotechnology, as previously detailed (Sutcliffe et al., 2009, 2011). Five micrograms of the following antibodies were used for ChIP: anti-PKC-θ (Santa Cruz), anti-Pol II (Abcam), and anti-LSD1 (Millipore). Immuno-complexes were enriched with Magna ChIP Protein A Magnetic beads (16-661, Millipore) and washed with ChIP wash buffers prior to DNA elution. Immuno-precipitated DNA was quantified using SYBR-green real-time PCR and normalized to total genomic input (2^−ΔCt^). Primers for human CD69 were:−241 to −52 relative to the TSS (Fwd: CCCACTTTCCTCCTGCTACA and Rev: GCCGCCTACTTGCTTGACTA) and +137 to +377 relative to the TSS (Fwd: CCGGAGAGTGGACAAGAAAG and Rev: GGGGTTTACCTCTTCCCTGA); and for the human miR200c promoter: (Fwd: CCACTGCCTTAACCCCTTC and Rev: AGGGGTGAGACTAGGCAGGT) previously described in reference (Wiklund et al., 2010).

### Bioinformatics

To help determine which transcription factors may be aiding the recruitment of PKC to DNA, we used the Genomatix program to examine the promoters of 69 of the top PKC binding genes. Our strategy was to look for transcription factor binding sites as defined by their TRANSFAC position weight matrices. The sequences of the PKC binding regions (±1 kb; Sutcliffe et al., 2011) were obtained from UCSC (Hg18). Match (Kel et al., 2003) was used to find where the over-represented motifs in the sequences occur with the core similarity score cut-off set to minimize the false negative rate and the matrix similarity score set to 0.9 for ZBPF, MAZF, MZF1, and SP1F members, 0.85 for EBOX and PAX5 members and 0.8 for E2FF, EKLF, and NFKB and 0.7 for AHRR members. The over-represented motifs belong to several superfamilies so to remove the redundancy when searching for co-occurring motifs, we reduced the set to NFKAPPAB_01 (NFKB), MYCMAX_03 (EBOX), E2F_03 (E2FF), MAZR_01 (MAZR), AHRARNT_02 (AHRR), PAX5_01 (PAX5), MZF1_01 (MZF1), and SP1_01 (SP1F). We calculated how frequently these motifs occurred together in groups of three within 300 bp. To determine if this was higher than by chance we also calculated the average number and SD of sequences that would have the three motifs within 300 bp if the motifs were distributed randomly among a similar number of sequences 1000 times. A co-regulatory motif was considered significant if it occurred more frequently than 2 SD above the mean of the 1000 random permutations. The calculations and visualization were performed in R.

### Computational modeling

The interaction between PKC-θ and the LSD1-CoREST complex was modeled using the ClusPro server (Kozakov et al., 2010), with the LSD1-CoREST crystal structure (pdb code 2IW5) and chain A from the crystal structure of the catalytic domain of PKC-θ (pdb code 2JED). The model shown in Figure 5C is the top ranked model.

### Protein kinase C sequence alignments

Protein sequences were retrieved from Uniprot for 11 of the PKC members (see Table in Materials and Methods for identification). By using Jalview (Waterhouse et al., 2009), combinations of nuclear localization motifs SPT, SPS, TPS, and TPT (termed SPT-like motifs) were identified. These motifis were displayed relative to the domains in PKC isoforms using CLC Main workbench 6 (CLC Bio). References demonstrating or predicting phosphorylation at the SPT-like sites are listed below.

**Table:** 

PKC isoforms	Uniprot name	Location	Sequence	Ref to show phosphorylation
PKC-δ	KPCD_human	240–242	SPT	
PKC-θ	KPCT_human	241–243	SPT	
PKC-ε	KPCE_human	337–339	SPS	
PKC-η	KPCL_human	317–319	SPT	Gauci et al. (2009), Mayya et al. (2009), and Oppermann et al. (2009)
PKC-α	KPCA_human	111–113	SPT	
		319–321	SPS	Beausoleil et al. (2006), Daub et al. (2008), Olsen et al. (2006), and Zahedi et al. (2008)
PKC-β (I)	KPCB1_human	111–113	SPT	
		642–644	TPT	Grodsky et al. (2006) and Zahedi et al. (2008)
PKC-β (II)	KPCB2_human	111–113	SPT	
PKC-γ	KCPG_human	110–112	SPT	
		326–332	SPSPSPT	
		326–332	SPT	
PKC-ζ	KCPZ_human	–	–	
PKC-λ/℩	KCPI_human	–	–	

### NLS

**Table:** 

PKC isoforms	Uniprot Name	Location	Sequence
PKC-δ	KPCD_human	613–625	KRRLEPPFRPKVK
PKC-θ	KPCT_human	644–656	RKEIDPPFRPKVK
PKC-ε	KPCE_human	678–690	QKKIKPPFKPRIK
PKC-η	KPCL_human	624–636	HRQIEPPFRPRIK
PKC-α	KPCA_human	607–619	NREIQPPFKPKVC
PKC-β (I)	KPCB_human P05771-1	610–622	RKEIQPPYKPKAR
PKC-β (II)	KPCB_human P05771-2	610–622	KEIQPPYKPKAR
PKC-γ	KCPG_human	624–636	RLEIPPPFRPRPC
PKC-ζ	KCPZ_human	528–540	KKQALPPFQPQIT
PKC-λ/℩	KCPI_human	552–544	QKQVVPPFKPNIS

## Conflict of Interest Statement

The authors declare that the research was conducted in the absence of any commercial or financial relationships that could be construed as a potential conflict of interest.
